# Development of Self-Healing Cement Slurry through the Incorporation of Dual-Encapsulated Polyacrylamide for the Prevention of Water Ingress in Oil Well

**DOI:** 10.3390/ma13132921

**Published:** 2020-06-29

**Authors:** G. Richhariya, D.T.K. Dora, K.R. Parmar, K.K. Pant, N. Singhal, K. Lal, P.P. Kundu

**Affiliations:** 1 Department of Petroleum & Energy Studies, DIT University, Dehradun 248009, India; gauravrichhariya@gmail.com; 2 Department Chemical Engineering, Indian Institute of Technology, Delhi 110016, India; kaushalrparmar@gmail.com (K.R.P.); kkpant@chemical.iitd.ac.in (K.K.P.); 3 Department of Chemistry, DIT University, Dehradun, 248009, India; drnaveen.singhal@dituniversity.edu.in; 4 Institute of Drilling Technology, ONGC, Dehradun 248003, India; kishorilal67@yahoo.com; 5 Department of Chemical Engineering, Indian Institute of Technology, Roorkee 247667, India; ppkundu1964@hotmail.com

**Keywords:** DUAL coated Polyacrylamide (PAM), self-healing cement, water absorption, expansion, microcrack

## Abstract

In the present work, a novel cross-linked polymer was synthesized though the anionic polymerization of cyanoacrylate with moisture as an initiator, methylene-bis-acrylamide as a cross-linker, and linseed oil as a spacer. Two layers of the synthesized polymer was coated over polyacrylamide for its homogenous impregnation in Class-G cement slurry for the synthesis of cement core. Fourier Transformation Infrared spectroscopy and X-Ray diffraction spectrum of the synthesized polymer and cement core were obtained to investigate the presence of different functional groups and phases. Moreover, the morphologies of the dual-encapsulated polyacrylamide was observed through scanning electron microscopy. Furthermore, the water-absorption capacity of the synthesized dual-encapsulated polyacrylamide in normal and saline conditions were tested. A cement core impregnated with 16% of dosage of dual-encapsulated polyacrylamide possesses an effective self-healing capability during the water-flow test. Moreover, the maximum linear expansion of the cement core was observed to be 26%. Thus, the impregnation of dual-encapsulated polyacrylamide in cement slurry can exhibit a superior self-healing behavior upon water absorption in an oil well.

## 1. Introduction

In oil well-cementing operations, ordinary Portland cement (API standard) is mixed with water and other additives such as dispersant, fluid loss additives, and friction loss additives to form cement slurry. The so-formed slurry is pumped downhole around the casing in an oil well. The cement sheath formed around the casing serves various functions such as supporting the weight of casing string, isolation of various zones around the casing and preventing the casing from oxidation. Often, cracks are developed in the cement sheath owing to shrinkage, de-bonding of the cement sheath from the casing or well-bore [1]. The generation and propagation of cracks usually depend upon the downhole stresses, temperature, and chemical reactions [2,3,4]. The crack generation in the cement sheath causes the migration of the formation fluids through the micro-fractures, thereby resulting in sustained casing pressure [5,6,7]. Upon crack development, the formation fluid starts migrating through the cement sheath into the well-bore, resulting in excessive water production or sometimes causes contamination of aquifers [8,9]. Therefore, the service life of the cementing can be increased either through crack-repairing by squeeze-cementing or application of self-healing cement. The squeeze-cementing technique was one of the most common methods for repairing the cracks in the cement sheath. However, the low success rate of 50%, excessive time consumption, and expensive nature of squeeze-cementing hiders its application now a days [10]. Moreover, the production needs to be stopped during the squeeze-cementing technique. Thus, self-healing cement can be an effective solution to overcome the issues related to squeeze-cementing for the prevention of water production in the oil well.

The autogenous self-healing technique is the inherent capability of the cementitious composite attributed to further hydration and carbonation [11,12,13]. The mechanism of autogenous self-mechanism is of two-fold such as hydration of unhydrated cement [14] and carbonation owing to precipitation of calcium carbonate [15]. During hydration, the tricalcium silicate (C_3_S) and dicalcium silicate (C_2_S) present in cement converts to C–S–H gel (calcium silicate hydrate phases) and portlandite (Ca(OH)_2_), thereby healing the crack [16]. However, Lapech et al. [17] investigated that the cracked cement concrete specimen did not possess any feature of autogenous healing of cracks during two weeks of saturation with water. On the other hand, the mechanism of carbonation is owing to the attack of the CO_2_ dissolved in water on the cement, thereby decomposing C–S–H phases into CaCO_3_ and silica gel, resulting in decrease in the cement strength [16]. Moreover, this intrinsic process may repair limited crack width up to 100 µm [18]. Therefore, chemical and biological amendment of the cementitious composite may result in better healing capacity. The self-healing capacity can be achieved through the incorporation of bacterial species into the cementitious matrix, as investigated by various researchers [18,19,20,21]. The water penetration through the cracks, activate the dormant microorganisms through precipitation of sufficient amount of the microbial calcite, in turn heals the cracks and thereby hinders the further ingress of water [18,22]. However, the bacterial species loses its healing capacity owing to higher pH of the concrete. Moreover, the spores of the bacterial species may get damaged because of the shear forces generated, shrinkage during mixing and drying of the concrete [23]. Thus, researchers have used diatomaceous earth as an efficient carrier for the transportation of bacteria. Diatomaceous earth immobilizes bacteria by precipitation of calcium carbonate though decomposing large amount of urea [24]. However, the water absorption by diatom during transportation limits its application. Thus, alginate hydrogel being an excellent immobilizer for bacteria though precipitating calcium carbonate was used by Belie [25]. The alginate hydrogel demonstrated good bacterial preservation along with good water absorption and moisture retaining capacity. However, the low compressive strength owing to the alginate hydrogel curb its use. Still various researchers have investigated the effect of bacterial incorporation in the cement matrix; however, the biomass viability test is yet to be explored [26]. Thus, the incorporation of abundantly available, chemically inert, durable, low toxic super absorbent polymers (SAP) in the cement matrix was emerged in the field of self-healing cementing applications.

The inherent hydrophilic groups of SAP are responsible for enhancing the interaction with the water molecules through hydrogen bond and the produced charged groups open the cross-linking of SAP, which in turn becomes the driving force of water absorption in terms of hundred times of its own mass [27]. Upon mixing with concrete, SAP forms a swollen hydrogel by absorbing and retaining water, thereby reducing the autogenous contraction and possible crack development [28,29]. Moreover, during the formation of microcracks in the cement sheath, SAP makes it water impermeable by virtue of its water-absorption capacity followed by swelling characteristics. The application of SAP for self-healing of cracks in the cement matrix was further explored by various researchers such as Lee et al. [30], and Snoeck et al. [31,32]. The impregnation of SAP by 5% (weight basis) in cement slurry resulted in a reduction of water flow through the 0.1 mm crack by 90% in 3 h [30]. The increased re-swelling ratio, particle size (500 µm) and reduction in initial swelling of SAP contribute in the improvement of self-healing cement [33]. However, the added SAP will swell by absorbing water in the cement slurry, thereby increases the viscosity, which in turn decreases the pumpability [28]. Moreover, if the dosage of SAP is less in the cement slurry, the plugging may not be effective. From the current literature review, the SAP in the cement slurry swells by absorbing water during its transportation into the oil well. Moreover, after placement of cement around the casing in the oil well, the SAP releases the water and shrinks, thereby leaving behind pores and thus decreasing the strength of the cementing. Thus, encapsulation of SAP can be an alternative method to isolate, protect, and control during its transportation along the cement slurry into the oil well. Therefore, Liu et al. [28] have encapsulated the SAP with multiple coatings of gypsum and chitosan before mixing with cement. However, the outer gypsum coating may erupt in basic solution, thereby exposing the chitosan layer, which may scrap away during pumping of the cement slurry.

Polyacrylamide (PAM) is high molecular weight water swellable, biocompatible polymers synthesized from acrylamide and its derivatives, thus widely used as a thickening agent, cross-linker, lubricant, and oil recovery agent. N–N methylene bis-acrylamide (MBIS) is a cross-linking agent used for polymer synthesis. Cyanoacrylate is a monomer of cyanoacrylate ester and the acryl group rapidly undergo chain-growth polymerization. Linseed oil being a spacer, controls the rate of reaction of cyanoacrylate during sudden exposure to moisture. Considering the fascinating characteristics of PAM, MBIS, Cyanoacrylate, and linseed oil in the present work, a dual-encapsulated of polyacrylamide was synthesized. The first layer of encapsulation comprises of methylene bis-acrylamide cross-linked with linseed oil and cyanoacrylate. Furthermore, another layer of fine silica, methylene bis-acrylamide cross-linked with linseed oil and cyanoacrylate was encapsulated. Moreover, the various dosage of dual-encapsulated PAM was homogeneously impregnated in cement slurry to prepare the cement core for its characterization. The water-absorption capacity of the synthesized polymers was investigated both in distilled water and saline water. Furthermore, the as-prepared cement core was split and performed for water-flow test to investigate its self-healing capacity. Moreover, the rheological behavior of the cement slurry with varying dosage of the dual-encapsulated polyacrylamide, were investigated to judge the pumpability through viscosity measurement. Simultaneously, the linear expansion against water and compressive strength of the synthesized cement core were measured, compared with the neat cement core to judge its applicability in the oil well-cementing.

## 2. Materials and Methods

### 2.1. Materials

Class-G oil well cement (API, High sulfate resistant), dispersant DO-65, and silica fumes were kindly provided by Institute of Drilling Technology, ONGC, Dehradun, India. DO-65 as a dispersant improves the rheological properties of the cement slurries. Moreover, it reduces friction pressure drop flow during pumping operations, thereby resulting in an improved placement which yields perfect zonal isolation across the zone of interest [34]. PAM (C_3_H_5_NO)_n_ was purchased from Sigma Aldrich Ltd., India. MBIS (C_7_H_10_N_2_O_2_) was purchased from Spectrochem India Pvt. Ltd. Cyanoacrylate was purchased from Rebecca polymers, India. Linseed oil (C_57_H_98_O_6_) was purchased from the local market. All the chemicals were analytical grade and used as received.

### 2.2. Synthesis of Dual Coated SAP and Cement Core

**Figure 1:**
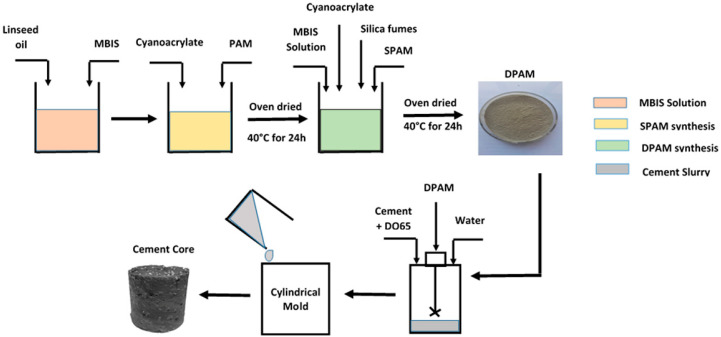
Schematic diagram for the synthesis of DPAM and cement core.

The schematic diagram for the synthesis of dual coated PAM and cement core is presented as Figure 1. Briefly, a weighed amount of MBIS was poured in linseed oil and then stirred in a magnetic stirrer at 300 rpm for 10 min to form a MBIS solution of 1:2 w/v ratio. A weighed amount of PAM (250 µm) was added to the MBIS solution followed by the addition of cyanoacrylate during stirring to obtain 1:3 w/v ratio. The formed viscous solution was stirred for 2 min to attain the first encapsulation of PAM. The obtained single coated PAM (SPAM) was dried in a hot air oven at 40 °C for 24 h. For the second encapsulation, SPAM, MBIS solution, and cyanoacrylate was mixed in a magnetic stirrer, with the further addition of silica fumes (SiO_2_) during stirring to obtain dual coated PAM (DPAM). The addition of silica fumes in the outer layer during DPAM synthesis helps in improvement of the bonding of PAM in the cement matrix along with a reduction in macropore formation [35]. The DPAM was again dried in the hot air oven (ACM 22064-I, AcMas Technologies Pvt. Ltd., Mumbai, India) for 24 h. Prior to the preparation of cement slurry, Class-G cement (Table 1) was sieved through 300-micron screen. Different weight percentages (4, 8, 12, 16) of DPAM, DO-65, and water were added to cement for the preparation of different samples of cement core (Table 2).

**Figure 2:**
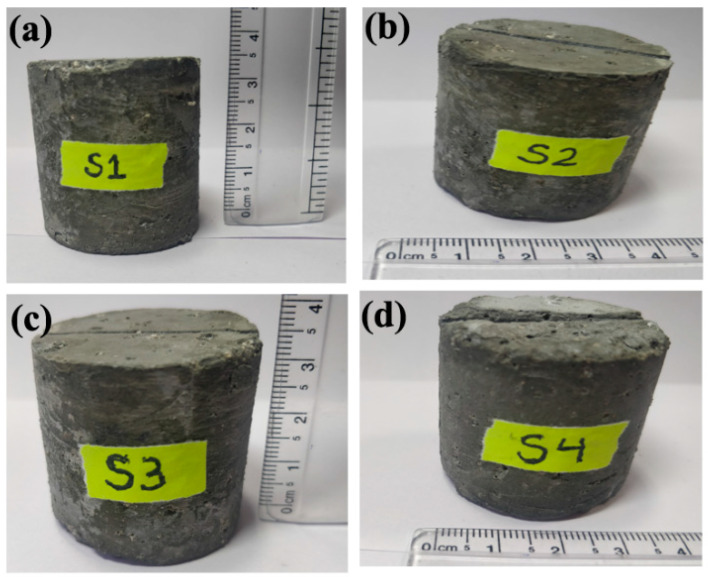
Snapshots of the synthesized cement core with (**a**) 4% (**b**) 8% (**c**) 12% and (**d**) 16% dosage of DPAM.

The mixture was blended in a warring blender at 12,000 rpm to obtain the cement slurry. The cement slurry was further poured in a cylindrical steel mold (37.3 × 38.5 mm^2)^ and allowed to set for 48 h for the synthesis of cement core. The snapshots of the different samples of cement core are presented as Figure 2a–d. During the synthesis of cement core, it was observed that beyond 16% dose of DPAM, the cement core loses its structural integrity.

### 2.3. Water-Absorption Test

The salinity of produced water in an oil well varies from fresh water to high saline water (300,000 ppm) with respect to depth of the oil well [36,37]. Furthermore, Anrey et al. [37] had investigated that the produced water of Gulf of Guiena contains salts of sodium and potassium. Thus, in this work, water-absorption tests [38] of PAM, SPAM, and DPAM were investigated in distillated water as well as in varied saline water of sodium and potassium. For this, 1 g of DPAM was treated with distilled water and different concentration of potassium chloride, sodium chloride solutions separately for 24 h at 1000 rpm. The samples were intermittently removed at every 1 h, vacuum filtered, and then weighed before carrying out the further water-absorption test. The water-absorption capacity was calculated as per Equation (1).

(1) $$w=\frac{m_{t}-m_{i}}{m_{i}}$$

where *w* is the water-absorption capacity (g/g), *m_i_* (g) and *m_t_* (g) are the weight of the DPAM at t = 0 and time t, respectively.

### 2.4. Characterization

Scanning electron microscopy (SEM) (FEI Quanta 200, ThermoFisher Scientific, New Delhi, India) was used to characterize the morphologies of the synthesized PAM, SPAM, and DPAM. The PAM, SPAM, and DPAM particles were spread over the carbon tape mounted on a SEM plate. The samples on the SEM plate was then coated with 2–5 nm of gold to make them conductive, before obtaining the SEM morphologies. The Fourier Transformation Infrared (FTIR) spectroscopy (NICOLET iS50, ThermoFisher Scientific, New Delhi, India) and X-Ray diffraction (XRD) (X’PERT PRO, PANanalytical, Mumbai, India) spectrum of the synthesized polymer and cement core were obtained. FTIR measurements were conducted in transmission model in which the scan resolution was 4 cm^−1^ and there were 64 scans averaged for each measurement. The XRD spectrum were obtained within the range of well-known cement material 2° to 90° at a grade of 0.02° increments of Cu Kα radiation, whereas for PAM, SPAM, and DPAM the 2θ values were kept in the range of 2° to 80°. Goniometer (Model 90 Pro Series, Rame-hart Instrument Co., Succasunna, NJ, USA) was used to measure the contact angle of the cement core.

### 2.5. Water-Flow Test through the Microcracked Specimen

**Figure 3:**
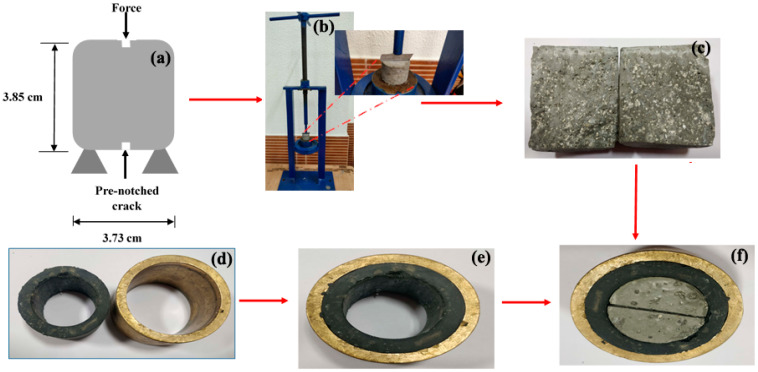
Schematic diagram of the cement core with a single penetrating crack for the flow measurement (**a**) pre-notched specimen subjected to crack generation (**b**) crack generation (**c**) snapshot of split specimen (**d**,**e**) core holder (**f**) assembled core with artificial crack.

**Figure 4:**
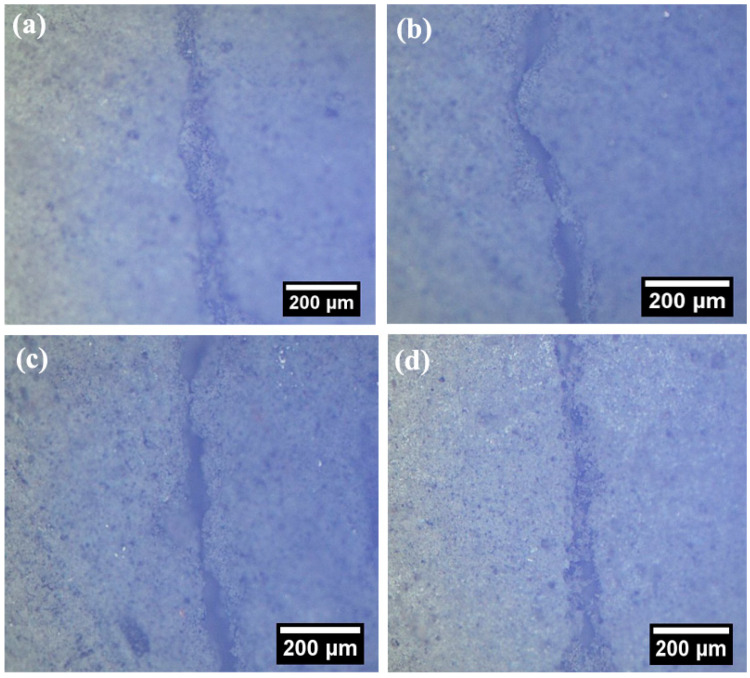
Stereomicrograph at the crack for the samples (**a**) S1 (**b**) S2 (**c**) S3 (**d**) S4.

For microcrack generation, first small groves of 1/8” depth on both sides of the sample were made exactly at the mid span along the diameter by tensile splitting using a loading device [33]. Sharp edged thin metallic plates were placed on each side of the grove and then pressure was applied at an increasing rate to induce a single through crack as shown in Figure 3b. The snapshot of obtained split samples is presented as Figure 3c. The morphology at lower magnification (4X) were obtained at both top and bottom part of the split samples using image analyzer (BX53M, Olympus, Hamburg, Germany) for measuring the crack width and presented as Figure 4. The crack width was measured by using image J software (1.53b, National Institute of Health, Maryland, MD, USA) at different positions for each sample. The average values of the crack width obtained are 73.07, 64.42, 62.65 and 70.24 µm for the samples S1, S2, S3, and S4, respectively. Thus, owing to the similar average crack width, these four samples were chosen for further analysis.

For water-flow test, cement permeameter (CGP-90, OFI Testing Equipment Inc., Houston, TX, USA) was used. The water was allowed to flow through the specimen (Figure 3f) under varied differential pressure ranging from 0.02 to 0.12 MPa. In this study, a 5 HP air compressor was used to generate the differential pressure across the specimen. Briefly, the split specimens were assembled and fastened in a brass specimen holder with a rubber sleeve arrangement (Figure 3d,e) to avoid any liquid leakage. With the system filled with water, the desired test pressure was applied to water reservoir and the initial reading of the gauge-glass was recorded. Simultaneously, a clean collection bottle was weighed and placed in position to collect the water percolating through the specimen. After every 4 h of continuous water flow the weight of the bottle was measured to calculate the mass flow rate through the specimen.

### 2.6. Investigation on Swelling, Rheological, and Mechanical Properties

The swelling properties of the prepared cement cores were investigated using an annular expansion mold [39]. Briefly, the cement slurry with additives was poured into the annulus between the two rings and allowed to set for 48 h. Then the mold was put in a water bath for 8 h. Upon absorbing water, the cement core swells thereby increasing the distance between the two slits in the outer periphery. The distance between the two slits was measured before and after swelling using a digital screw gauge, then the linear expansion was calculated by Equation (2).

(2) $$Linearexpansion=\frac{\left(M_{t}-M_{i}\right)}{M_{i}}$$

where *M_i_* and *M_t_* are the micrometer measurements at initial and at time “t” respectively.

The rheological parameters viz. viscosity and shear stress of the cement slurry with a varying dosage of DPAM was investigated using a rheometer (MCR-72, Anton Par, Graz, Austria). Briefly, the as-prepared DPAM impregnated cement slurry was placed in the coaxial cylinder of the rheometer, then the temperature was adjusted to 40 °C. The sample was then subjected to a stepped ramp of 5 s^−1^ and the viscosity measurements were conducted at 20 different shear rates ranging from 5 to 400 s^−1^. For the investigation of compressive strength, different dosages of DPAM were mixed in the cement slurry as mentioned in Section 2.2, poured in a cubical shape mold (2 × 2 × 2) and then cured at 50 °C for 24 h. The compressive strength of the prepared cubical specimen was measured using Compression Testing Machine (AIM 320-AN CTM, Aimil Ltd., New Delhi, India).

## 3. Results and Discussion

### 3.1. Polymer Synthesis Mechanism

**Scheme 1:**
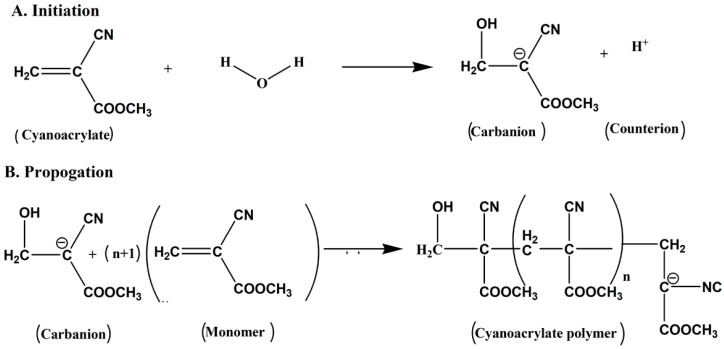
(**A**) Initiation (**B**) Propagation reaction of polymer synthesis.

**Scheme 2:**
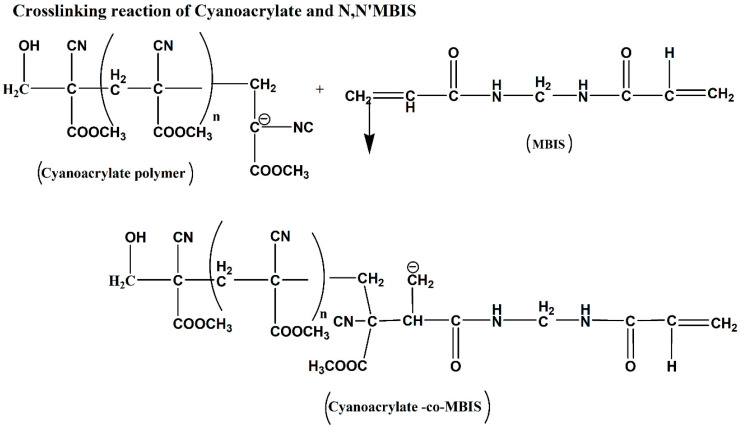
Cross-linking reaction of cyanoacrylate and MBIS.

**Scheme 3:**
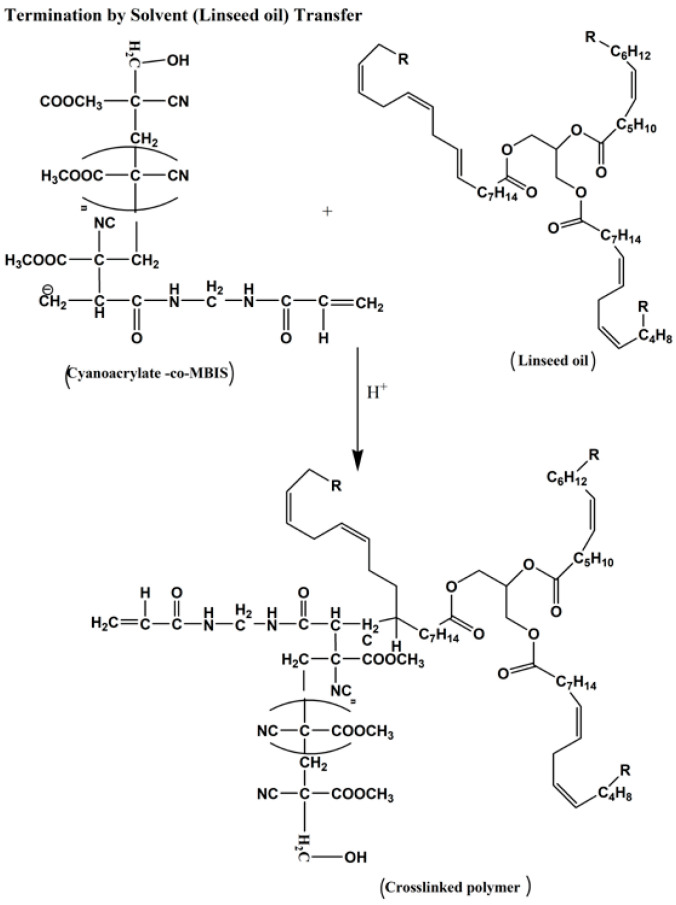
Termination by solvent (linseed oil) transfer.

The plausible mechanism for the synthesis of the novel cross-linked polymer is presented as Scheme 1, Scheme 2 and Scheme 3. In the first step, the moisture through lone pair of oxygen attacks the double bond of the cyanoacrylate monomer to generate a carbanion (Scheme 1A). Furthermore, during propagation, the initiated carbanion monomer reacted with other cyanoacrylate monomers in rapid succession to generate cyanoacrylate polymeric carbanion (Scheme 1B). The polymer with active carbanion was then reacted with the cross-linker MBIS resulting in shifting of carbanion to the MBIS molecule (Scheme 2). Furthermore, MBIS is helping in cross-linking of cyanoacrylate polymers by adding another propagating cyanoacrylate polymer to the other acrylamide of MBIS. The generated carbanion on the MBIS molecule can be shifted to the abundantly available linseed oil molecule (Scheme 3). The termination of the cross-linking reaction occurred by the transfer of the anion to the linseed oil, and then neutralized by the addition of H^+^ generated in the initiation step. The addition of the cross-linker increases the solidity in the synthesized polymer. The linseed oil also acted as a spacer resulting in the reduction of rate of reaction of cyanoacrylate as it does not allow moisture to penetrate and thereby controlling the initiation step.

### 3.2. Characterization of PAM, SPAM, and DPAM

#### 3.2.1. FTIR Analysis

**Figure 5:**
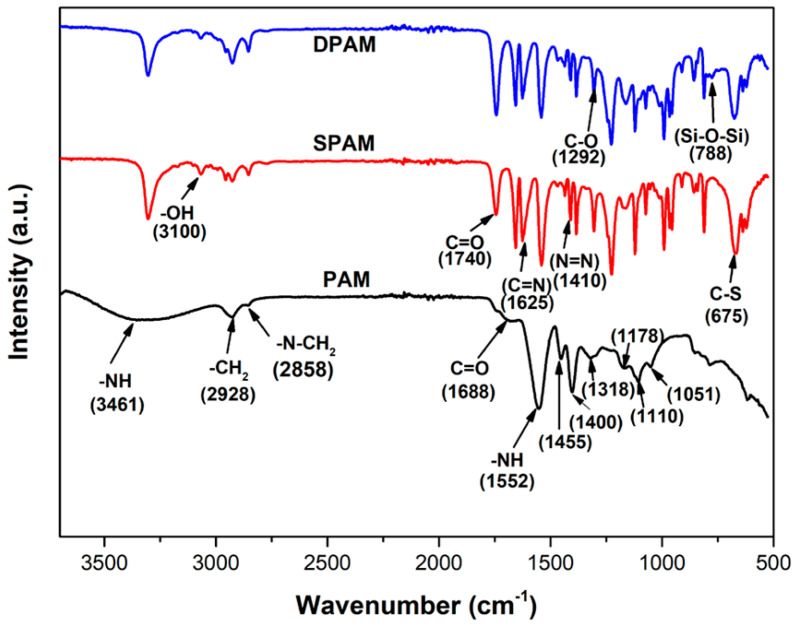
FTIR spectrum of PAM, SPAM, and DPAM.

The FTIR spectrum of the PAM, SPAM, and DPAM (Figure 5) confirm the presence of various transmittance bands and functional groups. The transmittance bands attributed to various functional groups in PAM was similar to the literature [40,41]. The NH groups appear at the transmittance band of 3461 cm^−1^ in PAM. However, during the synthesis of SPAM and DPAM, the transmittance band for NH groups was observed to shift from 3461 cm^−1^ to 3307cm^−1^. This is due to the cross-linking reaction of cyanoacrylate and MBIS (Scheme 2). In PAM, the band at 2928 cm^−1^ was attributed to asymmetric CH_2_ groups, which were also observed for SPAM and DPAM. Distinctive peaks were observed for PAM, SPAM, and DPAM at a transmittance band of 2858 cm^−1^, which was assigned to the –N–CH_2_ bonds. The intense transmittance bands at 1688 and 1552 cm^−1^ were observed in the synthesized polymers assigned to C=O owing to the termination of the polymer synthesis reaction through linseed oil (Scheme 3), the inherent NH bonds of the amidic group. The transmittance bands between 1400 and 1000 cm^−1^ were attributed to the presence of –C–N-links and CH_2_ groups. The polycyanoacrylate (Scheme 1) in SPAM and DPAM causes transmittance bands at 1625 and 1410 cm^−1^, which was assigned to C=N groups [42]. In SPAM and DPAM, a distinctive transmittance band also observed at 725 cm^−1^, which was attributed to the –CH_2_ wagging owing to the coating of the linseed oil (Scheme 3). In DPAM, the bands at 937 and 1051 cm^−1^ confirms the presence of Si–OH stretching and Si–OH rocking, owing to the coating of silica. The band at around 788 cm^−1^ is allocated to Si–O–Si cross-linking bonds [43].

#### 3.2.2. XRD Analysis

**Figure 6:**
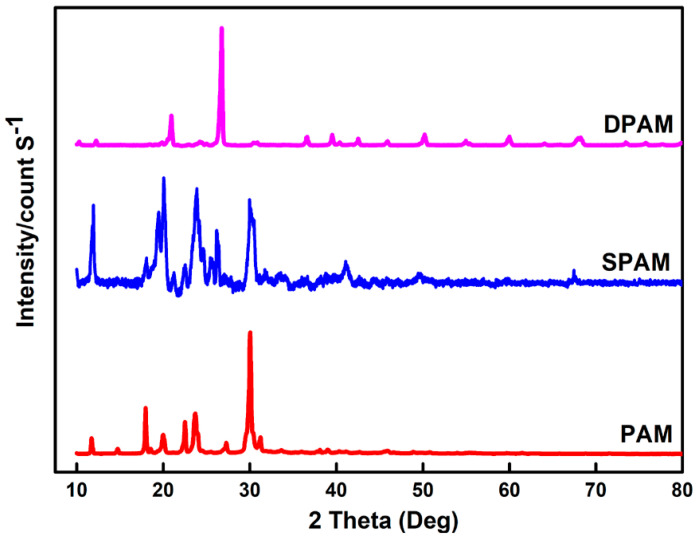
XRD pattern of PAM, SPAM, and DPAM.

XRD analysis was conducted to investigate the crystalline structure and phases present in PAM, SPAM, DPAM (Figure 6). In Figure 6, the XRD pattern of PAM showed intense peaks at 2θ values of 11.6°, 19.7°, 19.9°, 24.5°, 29.5°, 29.8° and 30.4°. These intense peaks reveal that the PAM used is crystalline amorphous in nature [44]. For SPAM similar intense peaks were observed at 2θ values of 23.86° and 41.09° owing to the encapsulation of MBIS. Furthermore, for DPAM along with the intense peaks of PAM and SPAM, other intense peaks at 2θ values of 49.6°, 59.9° and 68.2° owing to the encapsulation of silica particles. The decrease in the intensity of the peaks for SPAM and DPAM is due to the disturbed even outline of the atoms during encapsulation.

#### 3.2.3. SEM Analysis

**Figure 7:**
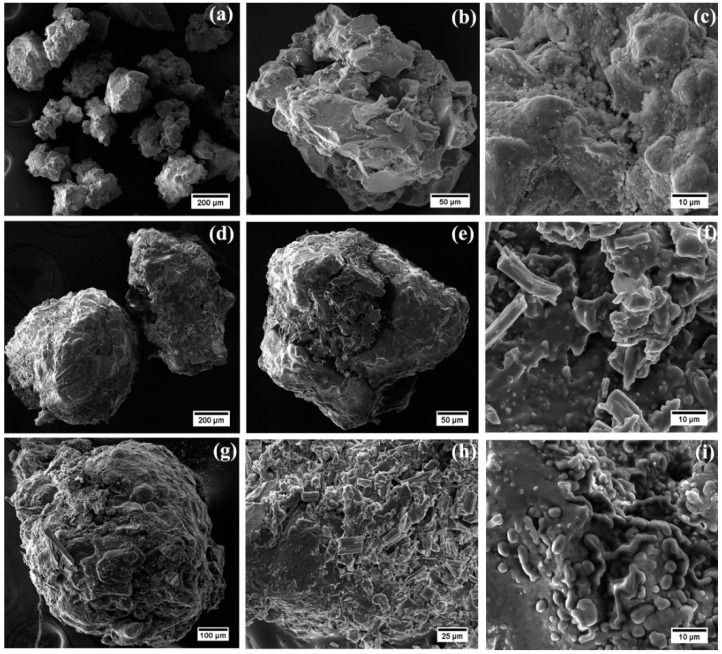
SEM micrograph of the synthesized (**a**–**c**) PAM (**d**–**f**) SPAM (**g**–**i**) DPAM.

The SEM micrographs for PAM at different magnifications are presented as Figure 7a–c. The average particle size of PAM chosen for dual encapsulation was found to be 224 µm (Figure 7a,b). Upon further magnification, cracks and crevasses were observed in the PAM particle (Figure 7b,c). The irregular shape and the observed cracks of the particles imparts an affirmative effect on the quality of encapsulation. The SEM micrographs for SPAM are presented as Figure 7d–f. After the first layer of encapsulation by cross-linked polymer, the size of the PAM particles was doubled, i.e., 542 µm (Figure 7d,e).

Upon further magnification (Figure 7f), a layer of synthesized polymer was observed at the outer layer of the SPAM particles. Simultaneously, the SEM micrographs for DPAM is presented as Figure 7g–i. The size of the particle after DPAM synthesis increased to 762 µm (Figure 7g) owing to the encapsulation of the second layer of cross-linked polymer embedded with silica fumes. The outer layer of the DPAM particles was observed to be flooded with silica particles (Figure 7h,i). The observed cracks in PAM particles (Figure 7b,c) were padded up through the migration of the cross-linked polymer and silica particles into the cracks during encapsulation. Furthermore, no cracks were observed in SPAM and DPAM particles (Figure 7f,i), confirming the attainment of the better encapsulation.

### 3.3. Characterization of Cement and Cement Core

#### 3.3.1. FTIR Analysis

**Figure 8:**
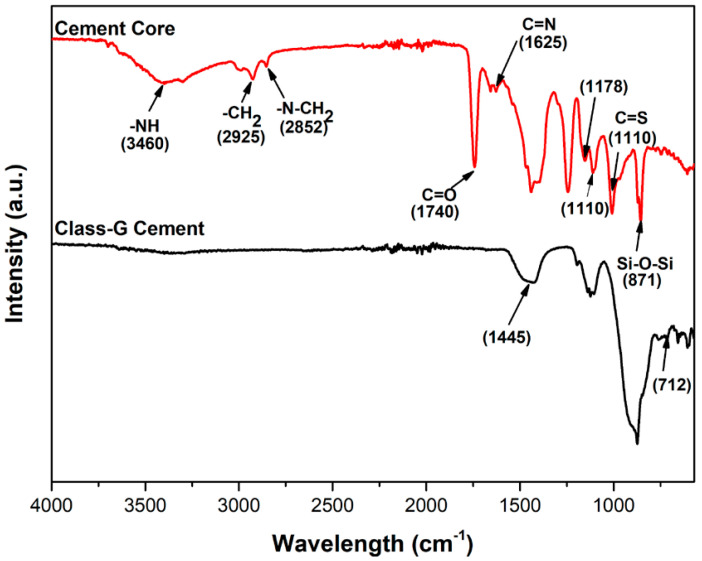
FTIR spectrum of cement and cement core.

Figure 8 shows the FTIR spectrum of Class-G cement and the synthesized cement core. A very weak peak at 3465 cm^−1^ assigned to symmetric and asymmetric O–H stretching. In both, the spectrum, the transmittance bands at 1445, 871 and 712 cm^−1^ were assigned to v_3_, v_2_, and v_4_ CO_3_^2−^, respectively [45]. The band at 871 cm^−1^ in the unhydrated Class-G cement was attributed to the stretching mode Si–O. The addition of DPAM in Class-G cement for the synthesis of the cement core caused the introduction of various functional groups in the cement core as discussed in Figure 5.

#### 3.3.2. XRD Analysis

**Figure 9:**
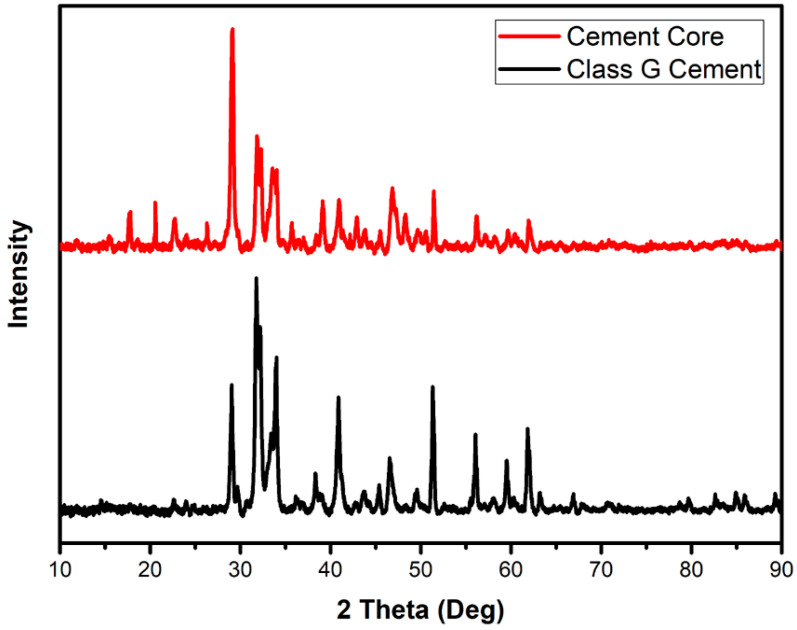
XRD pattern of cement and cement core.

Similarly, the XRD pattern of Class-G cement and cement core is presented as Figure 9. For Class-G cement the intense peaks at 2θ values of 29.48°, 32.24° and 32.42° indicate the presence of phases of tricalcium and dicalcium silicate. Furthermore, some overlapped peaks at 32.24° and 34.42° were observed owing to the presence of C_2_S (Ca_2_SiO_4_). Moreover, cement upon hydration produces hydrated calcium silicate (Ca_2_SiO_5_H_2_) which has an intense peak at 2θ = 29.48° and Ca(OH)_2_ at 2θ = 41.6° [46]. Simultaneously, in the XRD pattern of cement core, other intense peaks in the range of 15°–25° were observed owing to the mixing of DPAM in cement paste. This causes an introduction of functional groups of the organic matter of DPAM (Figure 9) and effects the cementitious properties of the cement in the mixture. The scattered DPAM in the cement paste yields a crystalline cement core owing to the chemical interaction of the various cement phase viz. C_3_S, C_2_S, and C_3_S with the polar functional groups of the organic polymer of DPAM.

#### 3.3.3. Contact Angle Measurement

**Figure 10:**
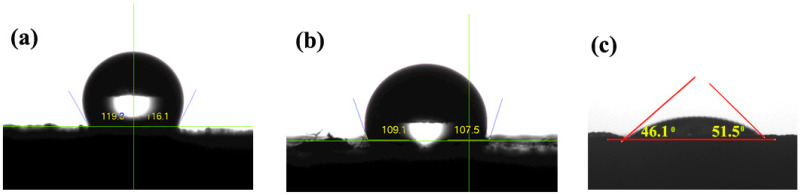
Water contact angle measurement of sample S4 at time of exposure of (**a**) t = 0 h (**b**) t = 1 h and (**c**) t = 5 h.

The snapshots of water contact angle measurement of the sample S4 is presented as Figure 10. It is observed that the water contact angle of 119.3° at the left and 116.1° at the right (Figure 10a), reveals that initially the sample was hydrophobic [47]. The change in contact angle between right and left side may be due to the surface irregularities of the cement core. After 1h of exposure with water, the average water contact angle is observed to decrease to 108.3° (Figure 10b). Further elapse of time at 6 h of exposure, the average water contact angle further reduced to 48.8° (Figure 10c), thereby revealing that the characteristic of the material changes to hydrophilic. This change in characteristics of the cement core sample suggests that the degradation of outer hydrophobic coatings had occurred within 6 h of exposure, thereby exposing the PAM to the water and in turn absorbing water.

### 3.4. Investigation of Water Absorption of PAM, SPAM, and DPAM

**Figure 11:**
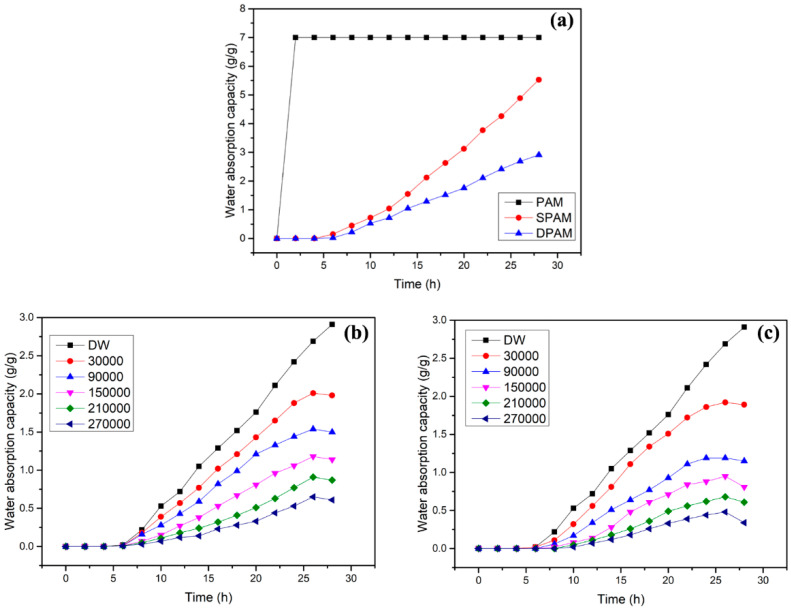
Water-absorption capacity of (**a**) synthesized polymers in distilled water (**b**) DPAM in KCl solution (**c**) DPAM in NaCl solution.

The water-absorption capacity of PAM, SPAM, and DPAM are presented as Figure 11a. It is observed that the water-absorption capacity of PAM is more than that of SPAM and DPAM. Moreover, the water-absorption capacity of PAM reaches its equilibrium value within less span of time, whereas for the SPAM and DPAM, the water-absorption capacity increases slowly with respect to time. The encapsulation of the MBIS and further silica over PAM hinders the direct exposure of PAM to water, thus decreasing both the water-absorption capacity and its rate. Furthermore, the detachment of outer layers over PAM over a period of time of 6h causes the migration of water into SPAM and DPAM, thereby increasing the rate of absorption and water-absorption capacity with respect to time of exposure. Simultaneously, the DPAM particles took a little longer period of time to swell as compared to SPAM owing to the hydrophobic characteristics of the outer encapsulated silica layer. The DPAM particles behave as SPAM after the detachment of the silica layer during stirring and swell further by absorbing the water.

Furthermore, DPAM was exposed to various concentrations (30,000 to 270,000 PPM) of KCl and NaCl solutions to investigate the water-absorption capacity in saline conditions as represented in Figure 11b,c, respectively. When DPAM was exposed to distilled and saline water, the water-absorption capacity remained constant over a period of 6 h, then the particles start absorbing water. It is observed from the figure that the water-absorption capacity decreases with increase in the concentration of KCl and NaCl solution. The increase in solution concentration causes in decrease in the polymer-solvent interaction, owing to the formation of electric double layers around the polar groups viz. C=O, C–N with the Na^+^ or K^+^ and Cl^−^ ions, thereby inhibiting the migration of water into the particles. Moreover, after prolonged use, the salt content wrench water from the particles owing to the osmosis effect, and thus the water-absorption capacity decreases.

### 3.5. Linear Expansion

**Figure 12:**
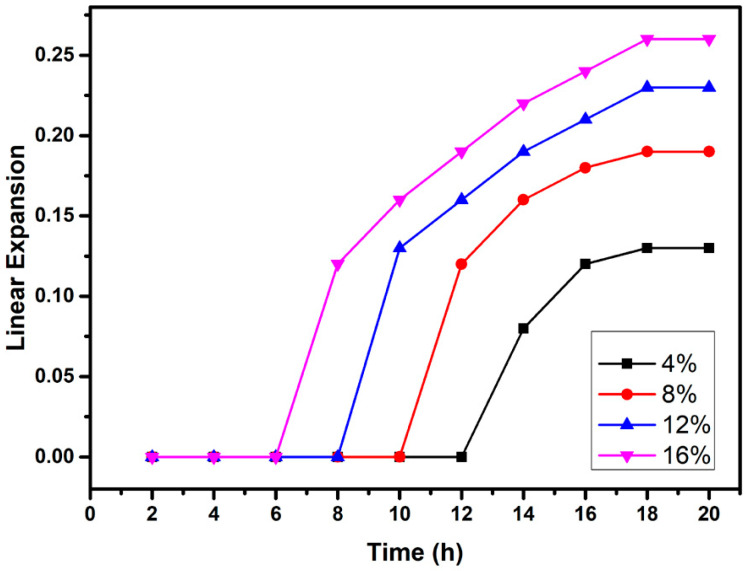
Linear expansion of cement core with different dosage of DPAM.

The linear expansion of the cement core doped with a various dosage of DPAM was continuously measured as a function of time over a period of 20 h and presented as Figure 12. It is observed that all the specimens did not expand in the initial hour, then the expansion increases exponentially with respect to time. The stagnant phase is owing to the dual encapsulation of silica and MBIS, after degradation of the outer coated layers the PAM get exposed and the cement core starts expanding by absorbing water. As the dosage of DPAM increases, the stagnation time decreases. This may be because with an increase in dosage of DPAM, the force imparted by the exposed PAM particles becomes more in 16% dosage as compared to 4% dosage. It is also observed that as percentage of dosage of DPAM in the cement core increases the linear expansion also increases. The increase in the concentration of DPAM causes more absorption water, thereby increasing the linear expansion. For 16% DPAM dosage, the cement slurry could attain a maximum linear expansion of 0.26 mm/mm.

### 3.6. Water-Flow Measurement

**Figure 13:**
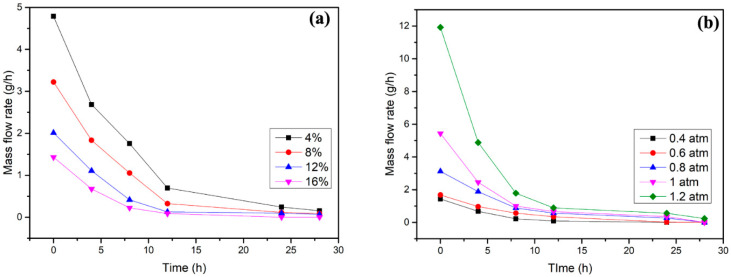
Water flow through single crack in the cement core for (**a**) various dosage of DPAM at 0.4 atm differential pressure (**b**) various differential pressures for 16% dosage DPAM cement core.

Figure 13a represents the dependence of water-flow rate on time of exposure at a differential pressure of 0.4 atm, through the synthesized cracked cement sheath with various dosage of DPAM. At the beginning the water-flow rate is maximum and as the time of exposure increases, the degradation of the outer layer occurs, thereby exposing the PAM particles. The exposed PAM particles absorb water and thus heals the crack, which in turn decreases the water-flow rate.

It is also revealed from Figure 13a that with an increase in the dosage of DPAM, the water-flow rate through the specimen decreases. It is observed that the specimen with 16% of DPAM ceases the water flow completely after 12 h of exposure owing to the self-healing of PAM. Thus, specimen with 16% of DPAM is chosen for the water-flow measurement for various differential pressure as represented as Figure 13b. As the pressure is increased the flow rate also increases according to the Hagen Poiseuille law [48].

**Figure 14:**
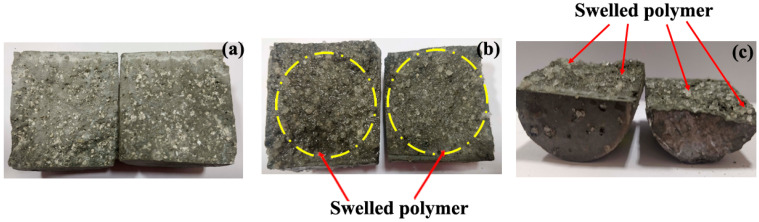
Snapshots of split cement core (**a**) before (**b**,**c**) after water absorption.

Figure 14 represents the snapshots of the cement core before and after absorption of water. It is observed that the polymer in the cement core swelled by absorbing water and thus heals the cracks generated in the cement core.

**Figure 15:**
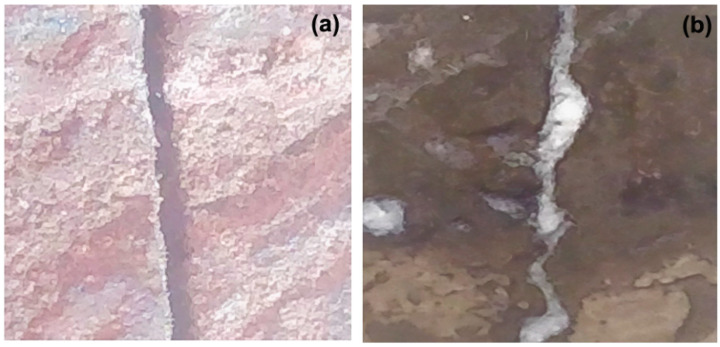
Snapshots of sample S4 (**a**) before (**b**) after water-flow test.

Figure 15 represents the snapshots of the cement core (S4) before and after water-flow test. It is observed that the generated crack was plugged after 12 h of continuous water flow.

### 3.7. Rheological Behavior

**Figure 16:**
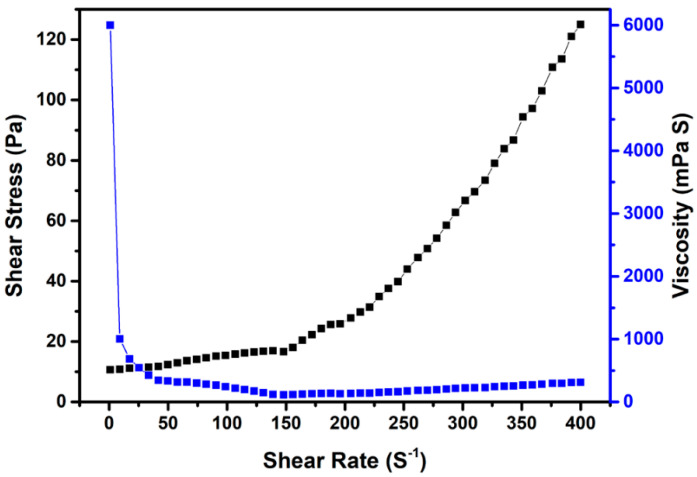
Rheological behavior of the cement paste.

The rheological behavior of the cement slurry with 16% dosage of DPAM is represented as Figure 16. From the shear stress vs. shear rate results, it is clear that the cement slurries behave like a Bingham plastic. Moreover, the variation of viscosity with respect to the shear rate is also presented in Figure 16. At the onset of the rheological study, the cement slurry tends to possess higher viscosity owing to its gelation characteristics. At the lower shear rate, the yield point of gelation of cement slurry breaks quickly and thus the viscosity drops drastically. Beyond yield shear stress, the viscosity tends to decrease at a lower rate owing to the deformation of the cement slurry. Further increase in the shear rate the viscosity tends to increase because of the further hydration of cement slurry to form a paste. Furthermore, there is not much variation in shear stress and viscosity found with respect to change in dosage of DPAM in the cement slurry.

### 3.8. Compressive Strength

**Figure 17:**
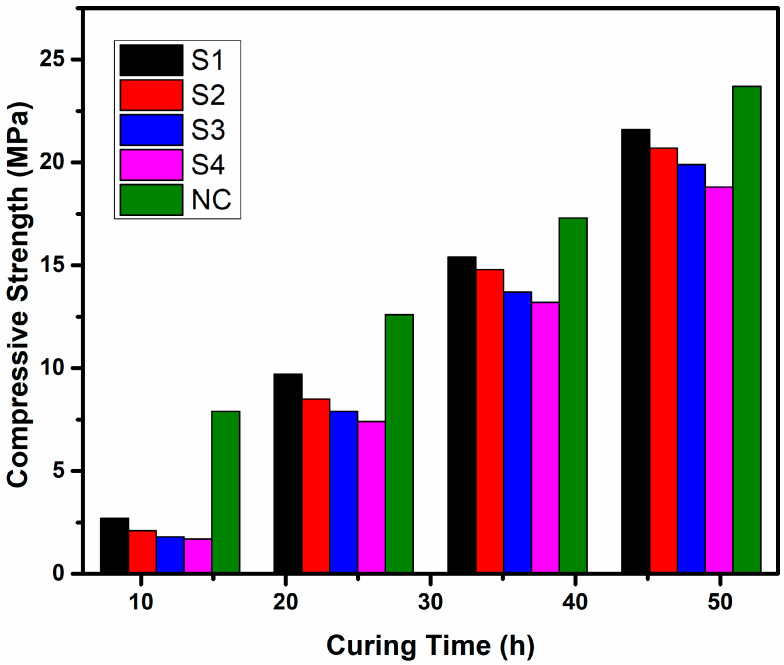
Compressive strength of cement core.

The compressive strength of cubical shaped cement core was measured using the compression test machine. The measured compressive strength of the specimens for various curing time is presented in Figure 17. It is observed that the compressive strength of the specimen decreases in a little margin with an increase in dosage of DPAM. The maximum compressive strength of 21.6 MPa was observed for the sample S1 after a curing time of 48 h and for the 16% DPAM cement core (S4) possess a compressive strength of 18.8 MPa.

Furthermore, a comparison of the linear expansion, water-absorption capacity and compressive strength of the as-prepared cement cores in this work with the other synthesized core reported in the literature is represented as Table 3. The difference in wate r-absorption capacity of polymer, linear expansion and compressive strength of the cement core are owing to the different types of polymer and material encapsulation chosen for impregnation. Furthermore, the water-absorption capacity owing to impregnation of SAP alone in the cement core is found to be maximum [49]; however such high absorption during the preparation of cement slurry before transportation into the oil well is undesirable. In the present work, the compressive strength of the cement core is in the concurrence.

## 4. Conclusions

To hinder the permeability of water and improve the stability of the hardened cement paste a dual coated water swellable polymer was synthesized and then impregnated in cement paste to enhance crack self-healing characteristics elicited by water leakage through the crack. Based on the experimental results the following conclusions may be drawn.

- The synthesized DPAM start absorbing water after 6 h of exposure, thereby increases its stability during cement slurry transportation into the oil well, thus justifies its impregnation in the cement slurry.
- The DPAM absorbs water about 250% of its own weight after 26 h time of exposure. Moreover, it can also absorb water about 40% of its own weight under a maximum oil well salinity of 270,000 ppm.
- The synthesized cement core with 16% dosage of DPAM possessed better self-healing capability within 12 h.
- With increase in dosage of DPAM in the cement core the linear expansion increases with a maximum of 0.26 mm/mm.
- The cement core with 4% dosage of DPAM has shown a maximum compressive strength of 21.6 MPa.

Thus, the novel dual polymer encapsulation prevents water migration into PAM during its transportation with the cement slurry into the oil well. Furthermore, upon crack generation in the cement sheath, the PAM plug the crack owing to water absorption instigating self-healing. Therefore, the cement slurry with 16% dosage of DPAM can be a potential cementitious material for preventing the produced water flow in the oil well with a little compromise with the compressive strength.

## Figures and Tables

**Table 1:** Composition of Class-G oil well cement.

Components	SiO_2_	CaO	Fe_2_O_3_	Al_2_O_3_	SO_3_	MgO	K_2_O	Na_2_O	Other
(wt.%)	22.84	63.72	5.02	3.46	1.69	1.30	0.46	0.89	22.84

**Table 2:** Mixing proportion of the cement paste.

Sl. No.	Specimen No.	Weight of Cement (g)	DPAM (wt.%)	DO 65 (wt.%)	Water (wt.%)
1	S1	500	4	0.2	44
2	S2	500	8	0.2	44
3	S3	500	12	0.2	44
4	S4	500	16	0.2	44
5	Neat Cement (NC)	500	0	0.2	44

**Table 3:** Comparison of the results with the earlier reported studies.

Sl. No.	Type of Cement and Additives	Water-Absorption Capacity of Polymer (G/g)	Linear Expansion of Cement (µm/m)	Compressive Strength (MPa)	Reference
1	Class-G Oil well cement, SAP, Calcium carbonate	12.5 (within 35 min)	Not studied	24.4	Liu et al. [23]
2	Cement, SAP	163	100–200	Not studied	Snoeck et al. [49]
3	Cement with fly ash, SAP	2.59	20	10.6	Lee et al. [33]
4	Cement, Fly ash, SAP	8 (after 2 h)	Not studied	15	Snoeck et al. [50]
5	SAP, poly-carboxylate ether, ordinary Portland cement	27.5 (within 15 min)	100	54	Baloch et al. [35]
6	Cement, chitosen, PAM, Class-G oil well cement	4.8 (within 5 h)	Not studied	38–41	Liu et al. [44]
7	Portland Cement, 5%SAP	0.08	Not studied	28.74 Mpa	Rai and Singh [46]
8	Class F Fly ash, CAC, sodium hexametaphosphate	Not studied	180	25	Sugama and Pyatina [51]
9	Epoxy acrylate, OPC, Cellulose	Not studied	900	7 (Flexural strength)	Lv et al. [52]
10	Class-G oil-well cement, DPAM	2.5 (after 26 h)	260	21.6	Present work
